# Supplementary material for: Evaluation of variability in performance and paw placement patterns by dogs completing the dog walk obstacle in an agility competition
Source: PLoS One. 2024 Mar 8;19(3):e0299592. doi: 10.1371/journal.pone.0299592 (PMC10923472; doi:10.1371/journal.pone.0299592)
Supplement: Attachment — Submitted filename: Major Revision - Response to Reviewers.docx [file pone.0299592.s003.docx]

Reviewer #1: Evaluation of variability in performance by dogs completing the dog walk obstacle in agility competition

It is a study that aims to establish the existence of well-defined paw placement patterns in dogs, with particular reference to an obstacle during agility trials.

The keywords seem adequate even if among them there is "sports medicine" in a way, in my opinion not very appropriate. In the study there is no observation of a clinical nature but only a visual evaluation of the gait.

**Author Response: Thank you for your comment. The authors are happy to adjust keywords based on recommendations from reviewers.**

The description of the study is very clear and articulated and the findings of the authors are well described in the "materials and methods" chapter.

Unfortunately, the study design has a very important limitation that cannot be overcome: the absence of any information, except for the height at the withers, of the dogs subjected to the study.
It is difficult, if not impossible, in the absence of data on race, age, conformation, morphotype, weight, to propose a study on gait and any classification into patterns as the elements for comparison are totally lacking.

**Author Response: Thank you for your comment. We agree it would be ideal to have more information regarding the individual dogs in each class. Unfortunately, this information is not available. The authors wanted to determine if there was merit to a larger prospective study where such information could be gathered by performing this initial evaluation. The authors respectfully disagree that it does not provide good starting information to be used in future studies.**

Similar previous studies relate the type of gait with the breed, with the "experience" (age) of the dog, while biomechanical studies establish that the gait mode is related to interactions between morphometric variables.

**Author Response: We appreciate your comment regarding breed and gait. While some studies do evaluate single breeds, there is also merit to evaluating dog populations as a whole when assessing the overall approach dogs use to complete obstacles. Later this can be assessed breed by breed and by varying conformation to determine if such differences alter gait patterns.**

My opinion is that the deficiency is decisive in assessing the suitability for publication of the article. Even if the authors honestly describe this limitation of the study (Discussion), I believe that the manuscript is not suitable for publication.

**Author Response: The authors respectfully disagree with the reviewer’s assessment that this data does not contribute to the literature regarding sporting dogs. The evaluation of gait in agility dogs in its infancy and basic studies such as this one must carried out and published prior to more in depth prospective studies. Preliminary data are also critical for providing the basis for funding mechanisms for future studies. Basic data such as this are the starting point for future, more involved studies, and direct the next phase of studies such as kinematic and kinetic evaluation.**

Reviewer #2: An original study of an agility obstacle seldom studied, however there is a preprint https://www.preprints.org/manuscript/201909.0158/v1 which should be referenced/cited in the article at intro and discussion.

**Author Response: Thank you for bringing this paper to our attention. The link in the pre-print paper you provided, there is a link to a “peer reviewed” version, unfortunately, this links to a completely different article. If you can direct us to the peer-reviewed version we would be happy to reference this article in the manuscript, however, as we were only able to find a non-peer-reviewed version, we have chosen not to include it at this time.**

I know that there is no way to go back and perform it again, but it would have been interesting to record if the dog has performed running, stopped contact or other to see if there was a difference between techniques on the variables studied. I see there is mention to this distinction at results, but not at methodology, so include this. Has any analysis been done to compare techniques?

**Author Response: Thank you for this suggestion. We were able to look at associations between observed contact behavior and paw placement patterns (and completion times) and have added this information to the manuscript; in the results (lines 178-185) and in tables provided as supplemental information.**

On statistics analysis, please inform software used and tests used to “explore differences by height of the dog”. For me, the main criticism is that it seems that stats have not been done to compare the variables by dog height. I recommend this to be done and reported.

**Author Response: Thank you for these comments and suggestion. We initially decided not to include formal statistical tests, as the primary focus of the study was descriptive and we did not have formal statistical hypotheses. However, in response to this feedback, we have added them to the manuscript with the caveat that they are exploratory in nature. We have updated the methods section (lines 124-127) and the results.**

**We have also added the statistical software used. (lines 130-131).**

Table 1 title is not correctly presented as it is not above the table but within the table

**Author Response: Thank you for this comment; this has been corrected.**

Table 1 should demonstrate the results of the stats to explore the differences by height

**Author Response: P-values have been added to Table 1 as well as the results (line 137)**

Same for table 2, it would be interesting to run stats maybe a multivariate LR to see if there was a difference in paw positioning, and how many foot strikes happened depending on the dogs height.

**Author Response: We have added p-values to Table 2 to indicate the results of testing for any difference by height category as well as updated the results. (lines 142-145)**

“Agreement between reviewers” vs “All videos were watched by the primary reviewer (JKD)”: there is no description in the methodology for the inter-rater agreement methods, not even for stats, just reviewer (singular) consistency is mentioned. Please clarify this in the methodology to allow replication.

**Author Response: We have reworded the statement in the methods about the reviewers to clarify that there was one primary reviewer who viewed all videos. Reviewer consistency was separately estimated (with no attempt to reconcile disagreements among reviewers). (lines 81-84). The statistics for how reviewer consistency was assessed are included in the statistical analysis section at the end of the methods (lines 128-130).**

“There was more variability in completion time (Table 1) and more
variability in the number of paw hits (Table 2) even among dogs from the same height category on the down ramp.”: was this tested or just observational? Is this possible to be tested, maybe a COV? At least for speed within each height category?

**Author Response: These comments are observational, based on the descriptive statistics. We have not added formal statistical tests here, as it was not even an exploratory aim of our original study. We have clarified the results to emphasize that these results are descriptive and not based on a formal statistical analysis. (lines 174-178)**

“As expected, the total number of hits with the front and rear feet was lower
for the taller dogs, particularly for the up and middle ramps. A majority of taller dogs completed the up and middle ramps with exactly two front and rear foot hits and a larger percentage of shorter dogs needed three (or more) hits to complete these two ramps” Was this statistically significant?

**Author Response: Thank you for these suggestions; yes, the difference in number of total hits was statistically different by height category (see revised table 2). We have updated the results section to reflect these tests. (lines 141-149).**

Table 3 and 4, please use “2 on 2 off”: instead of 2o2o

**Author Response: This change has been made to both tables.**

For me the category 12.75-17.5 was the odd one out but without stats we cannot really be sure.

**Author Response: We have added the formal statistical test for a difference across categories by height class to both tables 3 and 4.**

Table 3 has a footnote with an * but no * on the table itself. Table 4 has an * but no footnote to explain what it means. I think you got both mixed up

**Author Response: Thank for noticing the errors tables 3 and 4. They have been corrected in the revised version**.

Methodology is incomplete, describe better the contact techniques (not everyone is familiar with them) as it just say: Information about contact zone performance was recorded. I imagine this meant the technique, so create descriptions here for each technique. Also expand on “paw hits on each ramp were also recorded”

**Author Response: We have added additional clarification to the methods to reflect the data we collected. Lines 108-116**
